# MPLA case: A proposal for purchasing SRS/SBRT QA equipment

**DOI:** 10.1002/acm2.70807

**Published:** 2026-09-24

**Authors:** Joel St‐Aubin, Alonso Gutierrez, Erica Kinsey, Cassandra Stambaugh, Dongxu Wang

**Affiliations:** ^1^ University of Iowa Health Care Iowa City Iowa USA; ^2^ Miami Cancer Institute Miami Florida USA; ^3^ CommonSpirit Health Mountain Region Centennial Colorado USA; ^4^ Tufts Medical Center Cancer Center Boston Massachusetts USA; ^5^ Memorial Sloan Kettering Cancer Center New York New York USA

**Keywords:** capital budgeting, managerial finance, MPLA case study

## Abstract

This work of fiction is part of a case study series developed by the medical physics leadership academy (MPLA). It is designed to generate discussion of the managerial and leadership challenges medical physicists can face. Through discussion and quantitative analysis, the case aims to teach learners basic finance concepts and build their ability to gather and analyze financial information for capital budgeting decisions. In this case, medical physicist Dr. Angel Allende is a junior physicist who started a new position at a cancer center with two linear accelerators. A recent upgrade outfitted one linear accelerator with state‐of‐the‐art capabilities for image‐guided stereotactic radiation therapy. Dr. Allende is tasked by the chief medical physicist to establish a new stereotactic quality assurance (QA) program at the cancer center. The physics team needs to prepare a business case for obtaining the necessary QA software and equipment. Unfortunately, Dr. Allende anticipates complications due to the existing budget appearing too low. Despite having never done capital budgeting before, Dr. Allende doesn't want to turn down this unique opportunity. This case study falls under the scope of and is supported by the MPLA, a committee in the American Association of Physicists in Medicine (AAPM).

**Learning Objectives (LO)^a^**

Teamwork & Collaboration, Problem‐Solving Skills

1. Understand the needs and constraints of purchasing new equipment.
2. Analyze the strategy used to develop the overall action plan for the relevant capital purchases.
3. Assess leadership and professional capacities required in developing a capital budget request.

Operation and Finance

4. Accounting and finance concepts: return on investment, operating income, cash flow, time value of money, pro forma financial statement. Understand that there are different payment rates for different treatment techniques.
5. Identify tools and resources necessary to develop a capital budget request.
6. With reasonable assumptions, develop a simple pro forma financial statement for this case, as a practice.

^a^The underscored terms are key words from MPLA Curriculum at https://w3.aapm.org/leadership/curriculum.php

## 1 INTRODUCTION AND CASE NARRATIVE

This work of fiction is part of a case study series developed by the Medical Physics Leadership Academy (MPLA). The intended use of this case, through group discussion or self‐study, is to encourage readers to discuss the situation at hand and inspire professionalism and leadership learning. This case study falls under the scope of and is supported by the MPLA, a committee in the American Association of Physicists in Medicine (AAPM). The quantitative example reflects accepted financial methods, but the data are illustrative. Users must perform their own due diligence when applying these methods clinically.

### 1.1 Part 1: The backstory

Dr. Angel Allende recently took a job as a radiation oncology physicist at the newly renovated Altitude Cancer Care (ACC) facility in the beautiful city of Rocky Springs. Angel was hired by the chief radiation oncology physicist, Blair Hallowell, MS, and was the junior of the facility's two staff physicists. Since arriving, Angel has spent most of the time getting used to the facility, staff, and technology. With previous work on a different vendor's technology platform, Angel was now faced with learning the nuances of a new linear accelerator (LINAC) and its associated software.

Radiotherapy services for the entire city have been traditionally provided by ACC, one other larger institution, and various single‐vault independent centers. There had been gossip within the community that the main competitor to ACC was pursuing a new strategy to increase their market share within Rocky Springs. The rumor was that they wanted to obtain a specialized radiotherapy system unique to the city and use this capital purchase to push a new marketing campaign: “Technology Cures Cancer.”

To remain competitive in the market, ACC recently replaced one of its old LINACS with one capable of image‐guided stereotactic radiation therapy. In addition to purchasing this new LINAC, ACC's director had aggressively worked to recruit a new radiation oncologist, Dr. Min Jiang. Dr. Jiang's personal goals were to quickly start a stereotactic program for treatment of cancers in the central nervous system (CNS) and the lung.

The new LINAC was configured with stereotactic capabilities, including high‐definition multileaf collimators, flattening filter‐free beams, and a kilovoltage (kV) imaging system. Before this, neither of the older LINACs had kV imaging. The acquisition of this highly technical linear accelerator presented many clinical opportunities for new treatment techniques while delivering efficiency improvements. However, it also brought challenges for the entire staff, as the therapists and physicists had no prior kV imaging experience and did not have the quality assurance infrastructure to support the new technology, especially when applied to a stereotactic program.

During the LINAC commissioning, Blair stopped by Angel's office.

“Hi Angel, you had kV imaging at your last job, right?”

Angel turned to face Blair. “We did. We had kV imaging capabilities on all of our machines and a suite of imaging protocols developed for specific treatment sites and imaging goals.”

Blair nodded. “So, I'm assuming you are fairly up to speed on the necessary quality assurance equipment for kV systems?”

Angel shrugged. “I was comfortable with the system and equipment we used, but it was a different vendor's platform.”

“Great!” Blair exclaimed. “As you know, we are moving right along with the commissioning of our new LINAC, and Dr. Jiang is eager to start a stereotactic program as soon as possible.” Blair paused before continuing. “Since you have the most experience of the group with kV imaging, I'd like you to take the lead in establishing the image guidance quality assurance program and procuring the necessary QA equipment and software for both standard treatments and Dr. Jiang's stereotactic program.”

Angel stared at Blair. Despite having plenty of experience with kV imaging, Angel didn't know the first thing about purchasing equipment or establishing new programs! After a pause, Angel stated, “Blair, I'm not sure I'm the best person for this. I know kV imaging, but I have no experience putting together a capital request for the equipment”.

Blair waved Angel off. “Oh, don't worry about that. The new LINAC came with a generic phantom, and we have $50,000 leftover to purchase any other equipment you need. Just put together a proposal for what equipment you recommend and a timeline for implementation, and we'll go from there.”

Fifty thousand dollars didn't sound like enough to Angel, but maybe Blair was right; perhaps it would be enough. Angel didn't want to say no to the first significant project assigned. If Blair thought Angel was a good fit for this project, Angel would trust Blair's expertise. Nodding to Blair, “OK. I can look into options and get you a proposal and timeline by next week.”

Blair grinned. “That's what I like to hear! I can't wait to see what you put together.”

The following week, Angel was a mess. Angel had spent most of the previous week and much of the weekend reviewing literature and guidelines, talking to former colleagues, and, at their suggestion, getting as many quotes for QA equipment as possible. Angel felt confident about the proposed QA program but was overwhelmed with the different equipment options and features. However, one thing was obvious. Fifty thousand dollars wasn't going to be enough for the equipment needed to set up an adequate quality assurance program for stereotactic radiation therapy. Angel knew for sure that they would need to request more capital.

Anticipating a tough conversation ahead, Angel attempted to put together a comparison of equipment, their associated costs, and the justification based on professional society standards. Angel hoped that the novice attempt would be enough. Then, Angel took a deep breath and headed to Blair's office.

“Come on in!” Blair exclaimed when Angel knocked on the door.

“Blair?” Angel started while opening the door, “I have my proposal on the stereotactic quality assurance program for you.”

“Great!” Blair pulled over a chair for Angel.

Angel sat down, “Well, first, I would like to address what I think is a pretty big issue.”

Blair's eyes narrowed. “Issue? What sort of issue?”

Angel proceeded to show Blair the researched options, the literature supporting the need for the QA equipment, and the associated costs. “Regardless of which equipment we choose, we will need an additional $75,000 to $100,000 to get up and going with this program.”

“We don't have access to that sort of funding right now!” Blair appeared disappointed. “The hospital just invested five million dollars into the new LINAC and the new vault. It is difficult to ask again. Are you certain all of this is necessary? What do you suggest we do?”

Angel winced. “In my opinion, we have two options. The first would be to purchase the bare minimum to get kV imaging going for standard treatments. We could do this without more capital, but we would have to delay implementing Dr. Jiang's stereotactic program until we could secure the additional funding.” Angel took a deep breath, “The second option would be to present a cost‐benefit analysis on the potential revenue loss by delaying the stereotactic program by one year. I also think we need to consider the potential dissatisfaction of Dr. Jiang who was recruited for this purpose. We could probably make back the extra money spent on QA equipment in a short time, I think.”

Blair stared at Angel, “Brilliant! I thought you didn't know how to put together a capital request! Could you show me the numbers?”

Angel hesitated, “Well, I am glad I am on the right track, but I only intuitively thought of this method; I didn't work out the details.”

“That is some great intuition you have then,” Blair interjected. “Yes, that is definitely the right track. In fact, this type of hypothetical revenue versus expense calculation is often done in our hospital for new clinical programs. We show it in a format called a pro forma financial statement.”

“Pro forma? ”Angel asked, “I might have heard about it before.”

“It's Latin for ‘as a matter of the form’,” Blair explained. “In a pro forma financial statement – sometimes just called pro forma for short – you produce a projection of future revenue, expense, and profit. It takes the same format as an income or cash flow statement; it is specific to the equipment needs for a new clinical program.”

“So, it's like making a prediction of the return‐on‐investment of the new capital investment,” Angel paraphrased.

“Exactly! Here the return would be the incremental profits due to our new stereotactic program, which is gained via the new QA equipment.” Blair confirmed.

Angel said, “I see. That's easy to understand. Although, the QA equipment is only a small part of the investment to enable the new program. Obviously, it's mainly the new LINAC that will make the program possible.”

“Indeed. But, at this moment everything else was already in place; they are the sunk costs and should be disregarded in our current decision‐making. Our decision‐making is about the investment in the QA equipment. We can construct the pro forma financial statement assuming the QA equipment alone being the capital investment.” Blair reassured Angel.

“Alright! How do I start then? Is there a template for a pro forma?” Angel is now getting eager to start working on this.

“Ah, you are using the term like a pro now!” said Blair getting equally excited. “You don't need a template for the financial statement itself; you can produce one yourself using Excel spreadsheets. Just look up some examples. You will need some numbers from me, including patient volume, payor mix, payment rates, and those sorts of things. I have them from the recent project for the new LINAC. I will send those over to you by the end of day today. You can then make reasonable assumptions to complete the pro forma.”

Angel remembered learning some basic accounting and finance concepts in college elective courses. “I should be able to put together a pro forma financial statement!” Angel felt confident.

### 1.2 Part 2: A guided quantitative exercise

#### 1.2.1 Review accounting and finance concepts

That evening, it took Angel a few hours to find the old college course binders at home. There, Angel read the basic accounting and finance concepts again.

After having to Google some concepts that were no longer obvious from the course notes, Angel realized both Wikipedia and Investopedia have clear and detailed explanations for the concepts needed. Many other investment educational websites have various articles on these basic concepts too.

Angel became re‐familiarized with the following types of financial statements:

- Balance sheet;
- Income statement;
- Cash flow statement.

Angel now had an idea of what to do; produce a ***pro forma* income statement** **1** for the next five years assuming the purchase of the QA equipment in Year 0. Angel initially had some difficulty deciding whether to use a *cash accounting* method or an *accrual accounting* method but decided that for the *pro forma*—as a matter of the form—it was reasonable to assume the two were the same. With that, Angel started a simple spreadsheet to itemize the revenues and expenses (Table 1).

**TABLE 1:** Angel's simple pro forma income statement outline.

	Year 0	Year 1	Year 2	Year 3	Year 4	Year 5
Revenue	?	?	?	?	?	?
Expenses	?	?	?	?	?	?
Net Income						

Angel knew that Blair's numbers were needed to estimate all those cells marked as “?” for now, and that net income was the subtraction of expenses from revenue, but that data had not arrived yet. Assuming those numbers were available, how could the net income from the subsequent five years be compared to the initial investment?

Angel recalled a concept called the time value of money, which included future value and present value.**2**

$$\begin{array}{cc} & \mathrm{Present}\;\mathrm{Value}\;\left(\mathrm{PV}\right)=\mathrm{Future}\;\mathrm{Value}\;\left(\mathrm{FV}\right)\\ & \;÷\;{\left(1+\mathrm{Discount}\;\mathrm{Rate}\right)}^{n} \end{array}$$

where *Discount Rate* is often taken from some risk‐free return rate, such as United States Treasury bond or bills, and *n* is the number of time periods to consider. For example, if the cash income in Year 5 is $100,000, and the current five‐year US treasury yield is 4.00%, then the present value of the $100,000 income from Year 5 would be:

$$\$100,000\;÷\;{\left(1+0.04\right)}^{5}=\$82,193.\;$$

The same conversion can be calculated for every year considered and added up. Angel would then have a **net present value (NPV)**, the difference between the sum of the present values of future incomes and the current investment. In the end, the simple *pro forma* income statement outline would look like this (Table 2):

**TABLE 2:** Angel's pro forma income statement outline, with present value calculation incorporated.

	Year 0	Year 1	Year 2	Year 3	Year 4	Year 5
Revenue	?	?	?	?	?	?
Expenses	?	?	?	?	?	?
Net income						
Present value (PV)						
Net present value (NPV)						
Internal rate of return (IRR)						

The “Internal Rate of Return” (IRR)^3^ could also be calculated which Angel knew hospital administration reviewed when making investment decisions. IRR is a *derived* discount rate that would make NPV = 0. This one is mathematically complicated, but Microsoft Excel has a built‐in formula for IRR, Angel found.

The use of net present value and internal rate of return makes sense, Angel thought. If the total future income converted to present value is less than the current investment (i.e., a negative NPV, or IRR smaller than the chosen discount rate), why would the business bother investing in this project? They would just park their money into US treasury to earn the interest. Conversely, when NPV is positive and IRR is larger than the chosen discount rate, it implies that this investment has a more favorable return than “doing nothing”. It can also be used to evaluate competing projects, if needed.

With a “ping” sound from Angel's email, Blair's data arrived.

#### 1.2.2 Calculate payment rates and current revenue

Angel was thankful to Blair who had already compiled and organized the data for a previous project, although the data was still a bit overwhelming. The necessary data seemed to be scattered everywhere! Appendix Tables A1, A2 have all the source information about patient volume, disease site mix, payor mix, and average payment rate. Angel also needed some basic assumptions; luckily Blair had already provided those based on administrative knowledge and wrote them in the notes and annotations for Appendix Tables A1, A2.

Angel derived the following payment rates based on Blair's data and information (Table 3).

**TABLE 3:** Angel's calculated average payment for each treatment technique.

	Conventional treatment (average 20 Fx per course)	SRS/SBRT treatment (average 2.4 Fx per course)
*Treatment planning (per course)*		
Public payor	$1800.00	$1800.00
Private payor	$6000.00	$6000.00

*Treatment delivery (per fraction)*		
Public payor	$462.95	$2871.54
Private payor	$2161.50	$6891.70

*Total for a typical course*		
Public payor	$11,059.00	$8691.70
Private payor	$49,230.00	$22,540.08
Average payor payment (45% public, 55% private)	$32,053.05	$16,308.31

Angel then calculated the current total payment per year for the 600 patients, without implementing SRS or SBRT.

$$\begin{array}{cc} & \mathrm{Current}\;\mathrm{Annual}\;\mathrm{Revenue}=600\;\mathrm{Pts}\times \;\$32,053.05/\mathrm{Pt}\\ & \;=\$19,231,830 \end{array}$$

Feeling excited, Angel texted Blair: “Hi Blair! Sorry to bother you so late. I want to double check one number when you have a moment. I calculated our annual hospital revenue from our current patient volume is $19,231,830. Does that sound right?”

“Impressive!” It took Blair no time to reply. “These first two digits are certainly correct; I heard them from the hospital finance meeting just recently. The digits behind the millions may or may not matter for this project; we will have to see in a sensitivity analysis. By the way, thank you for working so hard and so late; I think it's time for you to take a rest. Have a good night!”

#### 1.2.3 Predict future patient volume mix

Angel continued the next morning while working from home. Having validated the method of calculating the current patient revenue, Angel just needed to project the future patient mix with SRS/SBRT and compute the future patient revenue.

As Angel reviewed the earlier work, something seemed off: the payment for a course of SRS or SBRT of $8691.70 appeared much lower than the payment for IMRT or conventional treatments of $11,059.00. How could that be? Was there an error somewhere?

After rechecking the calculations and obtaining the same numbers, Angel felt unsure, and texted Blair again.

“Hi Blair! I'm sorry to bother you so early but I have another question related to the revenue per patient.” Angel wondered if this was going to be a dumb question.

“What's your question?” Blair again replied back immediately. Angel couldn't tell if Blair was eager or agitated.

“Based on the payment rates you sent me, a course of SRS or SBRT would receive less payment than a course of IMRT or conventional treatment. Is that real?”

“Ah, that!” Blair replied seeming to anticipate this question already. “It's surprising and maybe counter‐intuitive, isn't? But it's true. Hypofractionation without patient volume increase is a net revenue loss for hospitals for this reason. We do it for the clinical benefits don't we?”

Angel took a deep breath of relief.

With this confirmation, Angel moved on to estimate the patient volume increase due to additional machine time freed up when some patients change from IMRT or conventional treatment to SRS or SBRT. Physicians had complained about the lack of treatment slots and one of them even suggested that, given a new machine, they would fill all its slots up. Thus, Angel would assume they would have no problem filling the new time slots. Angel needed some additional data to make the calculation, such as the typical time length of current IMRT or conventional treatment, as well as future SRS and SBRT treatment. These were nowhere to be found in Blair's email, however. Maybe this was the time to come up with reasonable assumptions. Based on real‐life experience, non‐SRS/SBRT treatment typically took 20 min for each fraction, while for an SRS/SBRT fraction would be about 36 min.

Blair's data already showed that eligible SRS/SBRT patients are estimated to be 12.68% of the current total. That is, 76 patients of the current 600 could be SRS/SBRT eligible. Based on Blair's data, 28% of these patients would be treated with single fraction SRS while the remaining 72% could be treated with 3 fraction SBRT. For simplicity, but without losing accuracy, Angel calculated the number of SRS/SBRT fractions would be 2.4 fractions / patient. With this assumption, Angel decided to calculate how many additional patients could be treated in the clinic with conventional fractionation if these 76 patients were treated with SRS/SBRT.

$$\begin{array}{cc} & 76\;courses\times \;\left(\;20\frac{fx}{course}\times \;20\frac{min}{fx}\;-\;2.4\frac{fx}{course}\;\times \right.\\ & \left.36\frac{min}{fx}\;\right)\;/\;\left(20\frac{min}{fx}\right)\;\;=1192\;time\;slots\;for\;\\ & IMRT\;or\;conventional\;treatment \end{array}$$

Assuming 20 fractions per conventional treatment, that's almost 60 additional patients—a 10% increase in patient volume! Angel understood that this was assuming all additional patients would be receiving IMRT or conventional treatment. The reality is some of them would also be SRS/SBRT eligible. The impact of this assumption would have to be left to the future sensitivity analysis

#### 1.2.4 Calculate future payment (revenue)

Angel was now ready to calculate the future patient volume mix, with 76 SRS/SBRT patients calculated from the information given in Table 1, and an additional 60 patients receiving conventional treatment (Table 4).

**TABLE 4:** Angel's calculated annual payment for future patient volume, when all current eligible SRS/SBRT patients are treated with SRS/SBRT, and an additional 60 patients are treated per year due to freed‐up treatment time slots.

	Conventional	SRS/SBRT
Future patient volume per year (660 total)	584	76
Average payment rate	$32,053	$16,308
Future annual payments	$18,718,981	$1,239,431
Future annual revenue	$19,958,413	

That's $726K more than the current annual payment! Angel jumped up with joy.

After getting a freshly brewed cup of coffee, Angel realized there was more work to do. Not only did this “future” state need to be prorated in a five‐year period time assuming ramp‐up, but it hadn't included the additional cost of operation yet.

Angel knew the QA equipment needed to be maintained which is why the quotes included a service contract. But what about the additional staffing needs? Blair was consulted again and shared, “Let's try $250,000 to start with. Add 3% each year. We can evaluate the other inputs in a sensitivity analysis.” With this information, Angel could estimate the annual expenses for the program. In addition to the staffing costs, Angel used the highest estimate for the cost of the additional QA equipment ($150,000) reasoning that if the *pro forma* looked good for this, it would look good for a smaller capital equipment request. Based on the QA equipment quotes, Angel knew that the annual services contracts would be 10% of purchase cost annually. Thus, Angel could fully estimate the annual expenses (Table 5).

**TABLE 5:** Angel's assumption about incremental operating costs (expenses) associated with the SRS/SBRT program.

*Incremental cost break‐down*	*Cost*	*Comments*
QA equipment	$150,000	One‐time; initial
QA Equipment service, maintenance & calibration	$15,000	From the vendor's quotes (10% of purchase cost); per year; locked.
Incremental staffing (Physics, RTT, dosimetrist, etc.)	$250,000	Per year with 3% annual increase.

#### 1.2.5 Construct a pro forma income statement for a five‐year period, and calculate the net present value

Angel wanted to project a five‐year ramp up from zero SRS/SBRT to full utilization as a means of estimating the annual incremental revenue for this project. For simplicity, the projected $726,583 additional income was linearly scaled over the five‐year period. Thus, Angel linearly increased the annual incremental revenue from 0 in yr 0, to the full $726,583 in year 5 (e.g., $726,583÷5=$145,317 in yr 1, etc.). Angel figured this was a reasonable assumption for the ramp up period. Angel now had an estimate of the annual revenue and expenses for each year. Angel then applied the concepts of the time value of money to calculate the present value of income in each year, the net present value, and the 5‐year internal rate of return (IRR). To do this, Angel used the current US treasury five‐year yield of 4.50% as the discount factor. With these, Angel constructed the *pro forma* (Table 6).

**TABLE 6:** Angel's five‐year pro forma income statement for starting the SRS/SBRT program with initial investment on the QA equipment.

Discount rate	4.50%	Discount rate was taken from the US Treasury 5‐year Note's yield around 5/6/2024.				
	Yr 0	Yr 1	Yr 2	Yr 3	Yr 4	Yr 5
Annual incremental revenue	$‐	$145,317	$290,633	$435,950	$581,266	$726,583

*Expenses*						
Capital expenditure	$(150,000)	$‐	$‐	$‐	$‐	$‐
Staffing	$‐	$(250,000)	$(257,500)	$(265,225)	$(273,182)	$(281,377)
Equipment service contract	$‐	$(15,000)	$(15,000)	$(15,000)	$(15,000)	$(15,000)
Total annual incremental expenses	$(150,000)	$(265,000)	$(272,500)	$(280,225)	$(288,182)	$(296,377)
Net incremental income	$(150,000)	$(119,683)	$18,133	$155,725	$293,084	$430,205
PV of net incremental income	$(150,000)	$(114,530)	$16,605	$136,461	$245,769	$345,219
NPV (5 year)	$479,524					
5‐Year IRR	37.8%					

*Note*: In accounting, negative values are often indicated by brackets “()”.

Abbreviations: IRR, internal rate of return; PV, present value; NPV, net present value.

The five‐year cumulative net present value pointed to a large return‐on‐investment, with an internal rate of return of an impressive 37.8%. Angel was ecstatic! Angel hoped that these numbers could convince the hospital administrators. At such a high IRR, Angel believed that the inclusion of the SRS/SBRT program would be a great return on investment (ROI)—the return the hospital would see for each dollar invested into the program.

The words of “sensitivity analysis” now rung in Angel's ear. Was this result too good to be true? What parameters should Angel vary to determine how sensitive the calculations were to the assumptions? If the total number of patients wasn't what Angel expected, that could change the results a lot! What about the assumed staffing costs? Angel also realized that despite the financial value for the implementation of the SRS/SBRT program, there were other non‐financial factors that could affect the overall decision. Although Angel didn't have much experience in this area, it was clear that other factors like patient quality of care, market positioning, operational capacity, staff recruitment challenges, and regulatory items that would need to be considered. However, for now Angel was simply happy to have a pro forma developed that showed the financial benefits of adding such an important program.

## 2 DISCUSSION

The case study presented is fictional and for education purposes only. Although this case study is intended to highlight main concepts related to managerial finance in the context of a realistic medical physics scenario, it is not necessarily a comprehensive list of items that need to be considered for capital budgeting purposes. This case study presents a feasible scenario that a medical physicist may encounter and presents concepts such as return on investment, time value of money, and pro forma financial statement. Additionally, it presents how different payment rates affect revenue. With all of these concepts, the case study also presents a workable example to help learners apply these concepts in a realistic scenario through the development of a pro forma financial statement.

The results of the presented case study rely heavily on the assumptions provided throughout the text. As an example, an IRR of over 37% is idealistic and only new additions to the program were included in the analysis. No equipment or program that was already in place was considered (i.e., they were considered sunk costs as part of this study as described in Part 1 The Backstory).

This case is in the scenario of Decision.^4^ The following are suggested discussion questions in relation to the Learning Objectives (LO):

1. On the technical side, many quality assurance guidelines are just guidelines and not requirements. How do you determine which tests must be performed at your department based on your equipment and program specifics, and how can you justify your decision? (LO1, LO2)
2. While it is nice to have new equipment and software designed for a specific task, many devices can be used in multiple ways. There are also acceptable home‐grown software solutions. Where do you draw the line between “critical to have” and “nice to have” when dealing with financial constraints? (LO1, LO2)
3. Different clinic members have different personalities and incentives. It is critical to learn how to advocate for appropriate software and equipment and lead the overall change. How do you approach this as the newest member of a team? (LO2, LO3)
4. Use spreadsheet software to replicate the quantitative work described in Part 2 (LO4, LO5, and LO6). An example spreadsheet is provided as a supplementary document titled ‘The supplemental document’. Learners should first complete the exercise independently and then use the example spreadsheet to compare and review their work. It should not be used as the primary resource for developing their spreadsheet.
5. Using the spreadsheet constructed from Question 4, now perform a sensitivity analysis by varying certain parameters or assumptions used in the calculation, such as the discount rate, service contract pricing, staffing numbers, and patient mix (SRS/SBRT patient numbers versus conventional patient numbers). A financial sensitivity analysis is a modeling technique used to determine how different values of an independent variable impact a specific output under a given set of assumptions. It helps analysts identify key risk drivers, test model robustness, and understand which assumptions most affect profitability or project value. ‘The supplemental document’ contains an example sensitivity analysis for the SRS/SBRT patient numbers. Learners should attempt the sensitivity analysis independently before consulting the example, which should be used to verify and compare their results rather than as a template for completing the exercise.

## 3 CONCLUSION

As listed in the Learning Objectives, this MPLA case encourages the readers to discuss and develop skills in two broad categories: (1) Teamwork, collaboration and problem‐solving, (2). Operations and finance. The case provided guidance and sample data in the Appendix for the readers to practice financial analysis and capital budgeting as a medical physicist may encounter a similar scenario as part of their work.

## AUTHOR CONTRIBUTIONS

Alonso Gutierrez, Erica Kinsey, Cassandra Stambaugh, and Dongxu Wang conceived the case study, and Joel St‐Aubin and Dongxu Wang developed the quantitative analysis study. All authors wrote the initial draft and contributed to revisions and final approval.

## CONFLICT OF INTEREST STATEMENT

The Chair of the Medical Physics Leadership Academy Cases Subcommittee (MPLACS) has reviewed the required Conflict of Interest statement on file for each member of MPLACS and determined that disclosure of potential Conflicts of Interest is an adequate management plan.

The members of MPLACS listed below attest that they have no potential Conflicts of Interest related to the subject matter or materials presented in this document.

*Joel St‐Aubin*

*Alonso Gutierrez*

*Erica Kinsey*

*Cassandra Stambaugh*

*Dongxu Wang*

## ETHICS STATEMENT

This case study is fictional and did not involve human participants, animal subjects, or any data collected from them. Therefore, ethical approval was not required.

## Supporting information



Supporting Information

## Data Availability

Data sharing is not applicable to this article as no datasets were generated or analyzed outside of the published text.

## 1

The following sample data and information can be used to build a *pro forma* financial statement to justify the proposed equipment budget. The reader may exercise reasonable assumptions when needed and does not have to add or modify any details provided, such as billing codes or payment rates. *The payment rates listed are hypothetical and simplistic, for the purpose of building a pro forma financial statement in this case study only*.

**TABLE A1:** Current payment volume by anatomical site and estimated SRS/SBRT eligible patient percentage.

Anatomical site	Percentage of total	SRS/SBRT‐eligible estimate as of the anatomical site
CNS (brain & spine)	24.0%	30.0%
Lung	8.2%	10.0%
Head and neck	8.8%	0.0%
Gynecological	8.4%	0.0%
Prostate and GU	10.0%	20.0%
Breast	21.0%	0.0%
GI (liver, pancreas, etc.)	7.0%	20.0%
Others	12.6%	10.0%
Total	100.0%	*12.68% of current patients*

*Note 1*: Metastasis occurrences are included in the respective anatomical sites.

*Note 2*: A total of 600 patients were treated on the 2 LINACs in the prior year. The current patient load is near the capacity for two LINACs, unless more plans become hypofractionated. We optimistically estimate that the local demand will fill the machine time capacity over time.

*Note 3*: Assume each SRS/SBRT course will have an average of 2.4 fractions per course of treatment, and non‐SRS/SBRT treatments have an average of 20 fractions per course.

*Note 4*: Public payors account for approximately 45% of the total patient volume, and private payors account for 55% of the total patient volume.

**TABLE A2:** Average payment rage for selected services.

Service/procedure	Public payor average	Private payor average
Treatment planning, per course	$1800.00	$6000.00
SRS or SBRT treatment delivery, per fraction	$2871.54	$6891.70
Conventional treatment delivery, per fraction	$462.95	$2161.50

*Note 1*: As of 2025, SRS, SBRT, IMRT, and non‐IMRT treatment all have different billing codes and different payment rates. This table produces a weighted average for simplicity.
